# Supplementary material for: PET Imaging of the Neurotensin Targeting Peptide NOTA-NT-20.3 Using Cobalt-55, Copper-64 and Gallium-68
Source: Pharmaceutics. 2022 Dec 6;14(12):2724. doi: 10.3390/pharmaceutics14122724 (PMC9781609; doi:10.3390/pharmaceutics14122724)
Supplement: Supplementary file 1 [file pharmaceutics-14-02724-s001.zip › pharmaceutics-2020349-supplementary.pdf]

Supplement for PET Imaging of the Neurotensin Targeting Peptide NOTA-NT-20.3 Using Cobalt-55, Copper-64 and Gallium-68

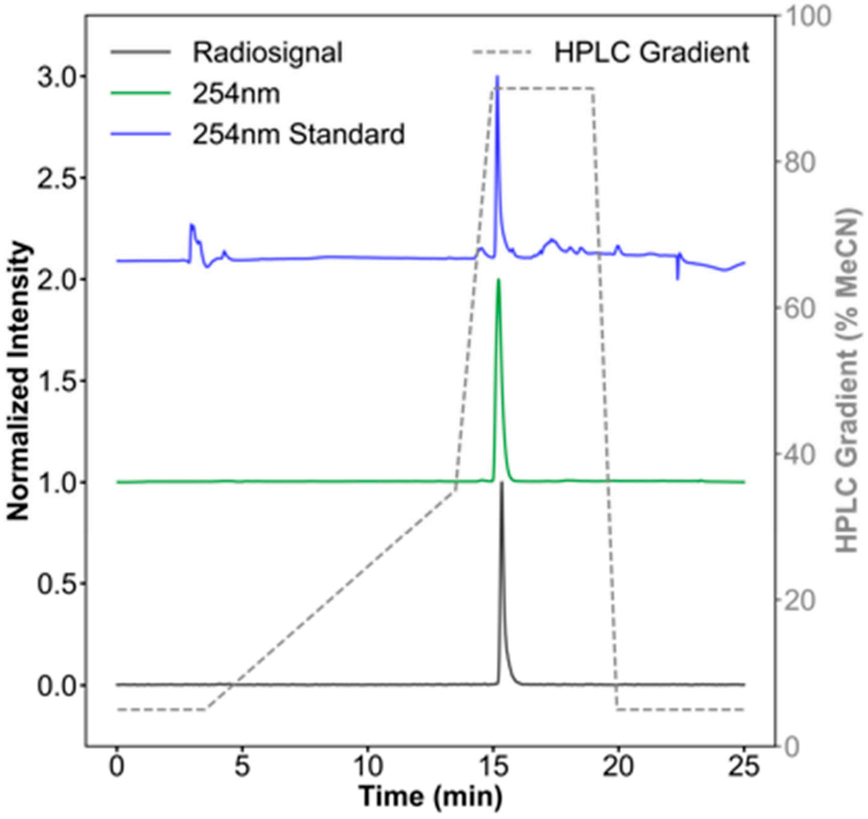

Figure S1. HPLC chromatograms of [^{55}Co]Co-NOTA-NT-20.3 (Radiosignal and 254 nm) and a Co-NOTA-NT-20.3 standard (254 nm standard). A graphical representation of the HPLC solution gradient (mixture of 0.1% TFA H_2O and MeCN) is shown in dashed lines.

A high specific activity method was also developed. [^{55}Co]Co-NOTA-NT-20.3 was radiolabeled at 7.4 MBq/nmol of ligand in pH 4.5 NaOAc buffer and heated at 95°C for 60 min with 2 mg/mL gentisic acid to inhibit radiolysis. Radiochemical purity was assessed by radio-HPLC using a reverse-phase 250 x 4.60 mm C18 5µm 100Å column (DIONEX) and the gradient outlined in figure 1. The HPLC data in Figure 1 show [^{55}Co]Co-NOTA-NT-20.3 radiolabeled using this method.

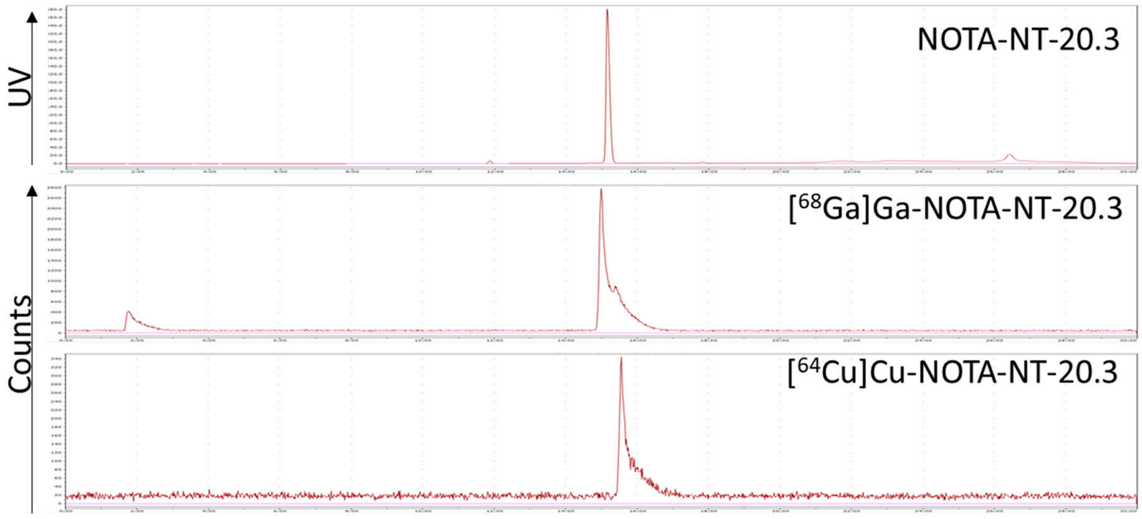

Figure S2. HPLC chromatograms of the [^{68}Ga]Ga-NOTA-NT-20.3 and [^{64}Cu]Cu-NOTA-NT-20.3.

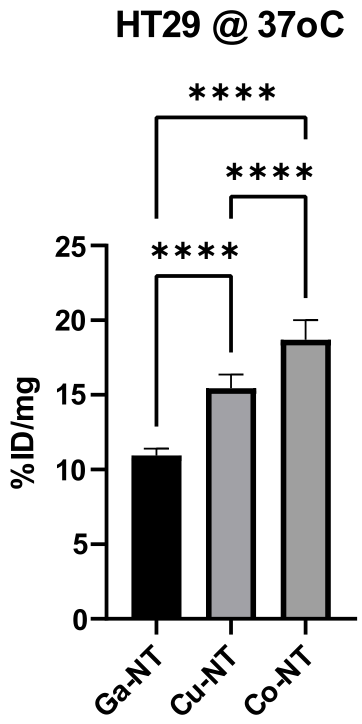

Figure S3. Statistical comparison of cell uptake of [^{68}Ga]Ga-NOTA-NT-20.3, [^{64}Cu]Cu-NOTA-NT-20.3, and [^{55}Co]Co-NOTA-NT-20.3 in HT29 cells. **** $p < 0.0001$.

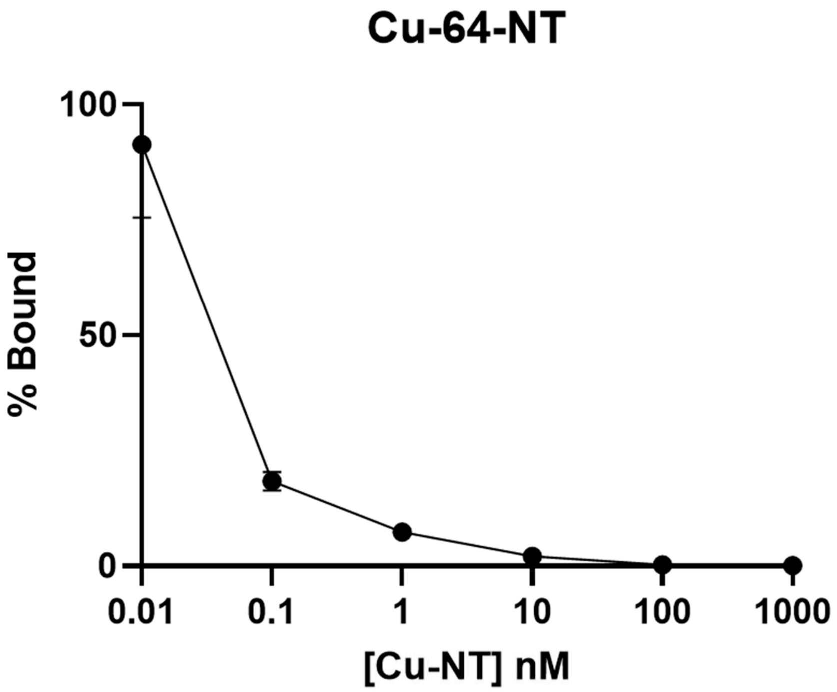

nM	0.01	0.1	1	10	100	1000
Cell CPM	45	50	150	550	750	2000
Total CPM	50	300	2500	25000	230000	1900000

Figure S4. Saturation binding of [^{64}Cu]Cu-NOTA-NT-20.3 shows that the IC50 is below 10 nM.
